# Course of Preexisting Migraine Following Spontaneous Subarachnoid Hemorrhage

**DOI:** 10.3389/fneur.2022.880856

**Published:** 2022-07-11

**Authors:** José Manuel Valdueza, Jens Peter Dreier, Johannes Woitzik, Christian Dohmen, Oliver Sakowitz, Johannes Platz, Stefanie Leistner-Glaess, Victoria Dorothea Witt

**Affiliations:** ^1^Neurological Center, Segeberger Kliniken, Bad Segeberg, Germany; ^2^Center for Stroke Research Berlin, Charité–Universitätsmedizin Berlin, Corporate Member of Freie Universität Berlin, Humboldt-Universität zu Berlin, Berlin Institute of Health, Berlin, Germany; ^3^Department of Neurology, Charité–Universitätsmedizin Berlin, Corporate Member of Freie Universität Berlin, Humboldt-Universität zu Berlin, Berlin Institute of Health, Berlin, Germany; ^4^Experimental Neurology, Charité–Universitätsmedizin Berlin, Corporate Member of Freie Universität Berlin, Humboldt-Universität zu Berlin, Berlin Institute of Health, Berlin, Germany; ^5^Bernstein Center for Computational Neuroscience Berlin, Berlin, Germany; ^6^Einstein Center for Neurosciences Berlin, Berlin, Germany; ^7^Department of Neurosurgery, Charité–Universitätsmedizin Berlin, Berlin, Germany; ^8^Department of Neurosurgery, Evangelisches Krankenhaus, Carl-von-Ossietzky University, Oldenburg, Germany; ^9^Department of Neurology, LVR-Hospital Bonn, Bonn, Germany; ^10^Department of Neurosurgery, Neurochirurgische Universitätsklinik, Heidelberg, Germany; ^11^Department of Neurosurgery, Heart-Neuro-Center Bodensee, Münsterlingen, Switzerland; ^12^Psychiatric Center Rickling, Rickling, Germany

**Keywords:** clipping, coiling, migraine, middle meningeal artery (MMA), subarachnoid hemorrhage (SAH), trigeminal nerve branches

## Abstract

**Background:**

Our objective was to observe the course of preexisting migraine following subarachnoid hemorrhage (SAH) in patients with and without craniotomy.

**Methods:**

We designed an exploratory analysis and hypothesis-generating study of prospectively collected data starting by recruiting patients suffering from SAH with the Hunt and Hess scale score of ≤ 4. Out of 994 cases, we identified 46 patients with preexisting active migraine defined by at least four attacks in the year before SAH. According to the treatment, we subdivided the patients into two groups: the first group included patients with surgical aneurysm clipping with transection of the middle meningeal artery (MMA) and accompanying trigeminal nerve branches and the second group included patients with endovascular aneurysm coiling or without any interventional treatment. During the follow-up, we recorded the course of migraine frequency, duration, intensity, and character.

**Results:**

For both groups (craniotomy *n* = 31, without craniotomy *n* = 15), a significant improvement regarding the preexisting migraine during a mean follow-up of 46 months (min. 12 months, max. 114 months) was seen regarding complete remission or at least >50% reduction in migraine attacks (*p* < 0.001 and *p* = 0.01). On comparing the groups, this effect was significantly more pronounced in patients with craniotomy (for no recurrence of migraine: *p* = 0.049). After craniotomy, 77.4% of the patients had no further attacks of migraine headache and 19.4% showed a reduction of >50% while only 2.2% did not report any relevant change. In the non-surgical group, 46.7% had no further migraine attacks, 20% had a reduction of >50%, while no change was noted in 33.3%.

**Conclusions:**

Our study provides evidence that the dura mater might be related to migraine headaches and that transection of the MMA and accompanying trigeminal dural nerve branches might disrupt the pathway leading to a reduction of migraine attacks. However, coiling alone ameliorated migraine complaints.

## Introduction

Migraine is generally assumed as a trigeminovascular headache syndrome. The current models describe vasoactive peptides such as calcitonin gene-related peptide (CGRP), substance P, vasoactive intestinal polypeptide (VIP), and pituitary adenylate cyclase-activating polypeptide (PACAP) being released in the trigeminal ganglion (1). According to these models, they reach vessels and the dura mater *via* efferent axons and lead to neurogenic inflammation with extravasation of plasma, upregulation of pain sensitivity, vasodilatation, stimulation of nociceptors, and activation of the trigeminal pain system.

Historically, Harold Wolff focused on the role of extracranial vasodilation in the pathogenesis of migraine headaches (2). However, most studies have concluded that extracranial vasodilation, if present at all, is an epiphenomenon rather than a key mechanism during a migraine attack and thus is no target for antimigraine drugs. For example, the potent vasodilator nitroglycerine was not effective in inducing an immediate migraine attack, whereas it immediately dilated the extracranial middle meningeal artery (MMA) (3, 4). Pain occurred only hours later when vasodilation had already subsided. Furthermore, the potent vasodilator VIP failed to induce a headache in patients with migraine (5) despite the fact that *in vitro* it dilates the human MMA (6).

Nonetheless, some more recently published studies support the notion that the MMA may be involved in the pathogenesis of migraine headaches through vasodilation and upregulated nociception. For instance, in 2010, Asghar et al. performed a double-blind, randomized, placebo-controlled, crossover study that included 18 healthy volunteers using magnetic resonance angiography (MRA). They found that CGRP caused the extracranial part of the MMA to dilate by 5.7% after correction for placebo-induced dilation without effect on the diameter of the main stem of the middle cerebral artery (MCA). Sumatriptan led to a vasoconstriction of the MMA in subjects not treated before with CGRP by 15% whereas the vasoconstriction of the MCA was only 5.3% (7). The *in vivo* studies in rats even showed that CGRP led to diameter increases of the MMA by more than 50% (8). Direct ictal MMA measurements are rare. However, in 2009, Nagata et al. performed MRA 2 h after the onset of a spontaneous migraine attack in a 42-year-old woman. The authors reported a diameter increase of up to 15% in the extracranial portion of the MMA compared to baseline (9).

There are rare observations about surgical options. In the 1930's, Dickerson followed up on the four patients with migraineafter MMA ligation, with three symptom-free (10). In a more recent study, ligation of the MMA together with the superficial temporal artery and the greater superficial petrosal nerve led to complete remission for at least 2 years in all the 10 treated patients with severe refractory migraine (11). Closure of the MMA using catheter techniques is nowadays used in the treatment of dural arteriovenous fistulas and chronic subdural hematomas (12, 13). Interestingly, seven out of nine patients with chronic subdural hematoma who suffered from “chronic headache” for at least 2 years and whose MMA was embolized were completely headache-free during a mean follow-up of 489 days (14).

The MMA is usually transected also during frontotemporal craniotomy in the surgical treatment of intracranial aneurysms. The aim of the present study was to investigate the course of preexisting migraine in patients with craniotomy following subarachnoid hemorrhage (SAH). Non-surgically treated patients with SAH without any intervention or coiling served as the control group.

## Methods

### Patient Recruitment and the Study Protocol

We recruited patients with acute SAH at a large center for neurological rehabilitation (CNR) and at six university clinics participating in Co-Operative Studies on Brain Injury Depolarizations (COSBID). In total, 763 patients entered the CNR with the primary diagnosis of SAH between March 2005 and January 2015. The selection of participants was performed by a detailed headache questionnaire comprising each clinical symptom as defined in the International Headache Society (IHS) criteria for migraine (15) when the patients were admitted with the principal diagnosis and after initial treatment of SAH. In addition, the six centers of the COSBID group enrolled 231 patients with SAH between April 2005 and March 2015. Prospective inclusion criteria for COSBID have been described previously (16). To analyze the impact of SAH and its treatment on the course of migraine headache, we screened patients with a Hunt and Hess scale score of ≤ 4 retrospectively for a history of active migraine prior to the SAH. All research was conducted in accordance with the Declaration of Helsinki. The Institutional Review Board approved the research protocols and we obtained surrogate informed consent from all the patients.

### Migraine

Migraine was diagnosed according to the criteria given in the 2^nd^ edition of the International Classification of Headache (IHS) Disorders (15). We defined active migraine as at least four migraine attacks within the year prior to SAH to rule out a warning leak as the harbinger of the SAH. We also screened for a history of migraine aura. The patients with only isolated aura but the absence of typical headaches were excluded.

### Subarachnoid Hemorrhage and Initial Treatment

Subarachnoid hemorrhage (SAH) was classified clinically according to the grading scale by Hunt and Hess (17). The amount and distribution of subarachnoid blood were graded according to the Fisher scale (18). After the diagnosis of SAH, surgical clipping and/or endovascular coiling was performed within the next 24 h. Secondary treatments (both coiling and clipping) were sometimes necessary due to a lack of effectiveness. Three patients did not undergo any interventional treatment due to failure to locate the origin of the bleeding. In all cases of surgical aneurysm clipping, a frontotemporal craniotomy was done, which includes transection of the MMA and the accompanying trigeminal nerve branches during the opening of the dura mater. In cases of surgical clipping, we documented the side of trepanation for correlation with the dominant location of migraine headache.

### Study Protocol

We selected all patients with SAH who suffered from preexisting migraine for subsequent analysis. For each patient identified with preexisting migraine, follow-up investigations (min. 12 months, max. 114 months) were performed by either a telephonic interview or an outpatient examination. In addition to the criteria for migraine and migraine aura, we collected the following data during initial and follow-up interviews: age, sex, prophylactic and acute medication prior and past SAH, location, and preferred side of headache. To address the issue of the transection of the MMA and trigeminal nerve branches, we compared patients with craniotomy and endovascular coiling or no treatment. For statistical analysis, we used the SPSS Statistics (IBM SPSS Statistics Version 20, International Business Machines Corp., New York, USA) software package. On analyzing the McNemar–Bowker test, the Wilcoxon test, and the Fisher exact test, we considered *p*-values of <0.05 significant. In accordance with standard prophylactic drugs, a significant reduction in migraine was defined as a decrease of more than 50% compared to the attack frequency prior to SAH (19). Possible confounders such as age, medication, changes in way of living, physical and mental condition, and stress factors were enquired in the interviews.

## Results

**Figure 1:**
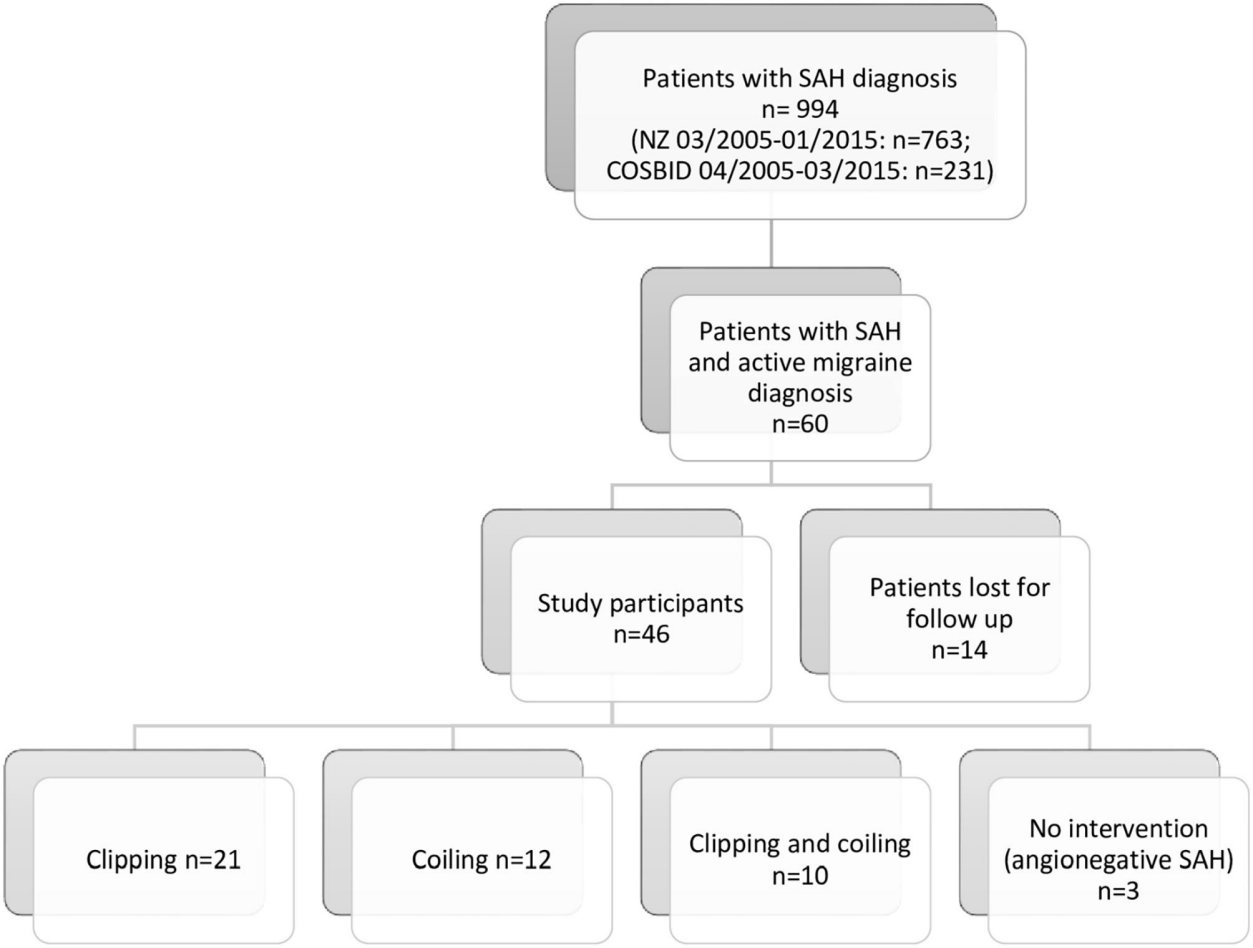
Overview of patient flow (NZ, center for neurological rehabilitation; COSBID, Co-Operative Studies on Brain Injury Depolarizations).

From 994 cases with SAH in the CNR and COSBID group (see Figure 1 overview), we identified 60 patients who strictly met the criteria from preexisting, active migraine (CNR *n* = 36, COSBID *n* = 24). Fourteen subjects failed to participate in follow-up investigations due to rejection, missing contact data, or death and, therefore, were excluded. Accordingly, 46 patients (see Table 1) were included in subsequent analysis and statistical calculation (CNR *n* = 33, COSBID *n* = 13). Table 2 summarizes the individual demographic and clinical data. Twenty-one patients underwent surgical clipping, 12 patients endovascular coiling, and 10 patients both procedures to finally treat the aneurysm. Three patients had angio-negative SAH. Fourteen patients had a history of migraine aura. Table 3 documents the migraine situation regarding frequency, duration, intensity, and attendant symptoms for the time before and after the occurrence of SAH.

**Table 1:** Table of demography — patient characteristics at baseline.

**Variables at baseline**	***N = 46***
Gender women/men, *n* (%)	38 (83)/8 (17)
Mean Age in years (SD)	47.6 (9.3)
Migraine diagnosis according to IHS criteria, *n* (%)	46 (100)
Migraine subtype without aura/with aura, *n* (%)	32 (70%)/14 (30)
Mean length of migraine diagnosis in years (SD)	23.9 (12.7)

**Table 2:** Basic characteristics of the included patients.

**#**	**Age**	**Sex**	**Aura**	**Migr.Atc/Y**	**Dom.** **Side**	**Aneurysm** **localization**	**Hunt & Hess**	**Intervention** **(Clipping/Coiling)**	**Side of** **trepanation**
1	52	Fem	N	24	Bilat	MCA	1	Clipping/Coiling right	Right
2	50	Fem	N	12	Vary	MCA	4	Clipping/Coiling left	Left
3	54	Fem	N	18	Bilat	ICA	3	Clipping right	Right
4	43	Fem	N	6	Bilat	ACA	2	Clipping right	Right
5	46	Fem	N	36	Right	MCA	4	Clipping/Coiling right	Right
6	47	Fem	N	30	Right	MCA	4	Clipping bilat.	Right
7	51	Fem	N	12	Right	Multiple	3	Clipping/Coiling right	Right
8	43	Fem	N	12	Right	MCA	2	Clipping right	Right
9	44	Fem	N	4	Right	MCA	3	Clipping right	Right
10	56	Fem	Y	24	Right	Multiple	4	Clipping left	Left
11	61	Fem	N	48	Vary	MCA	3	Clipping/Coiling left	Left
12	39	Fem	N	12	Right	AcomA	2	Clipping bilat.	Left
13	42	Fem	N	12	Left	AcomA	2	Coiling right	No
14	55	Fem	Y	42	Vary	MCA	3	Coiling left	No
15	45	Mal	N	72	Vary	No Ay	1	No intervention	No
16	51	Mal	N	12	Bilat	AcomA	2	Coiling bilat.	No
17	51	Fem	N	10	Vary	ACA	2	Coiling left	No
18	31	Fem	N	12	Vary	ICA	2	Coiling right	No
19	48	Fem	Y	12	Right	MCA	1	Clipping right	Right
20	47	Fem	N	4	Bilat	AcomA	4	Clipping left	Left
21	47	Fem	N	48	Vary	MCA	3	Clipping left	Left
22	34	Mal	Y	18	Vary	No Ay	1	No intervention	No
23	20	Mal	N	24	Right	No Ay	3	No intervention	No
24	47	Fem	N	6	Bilat	ICA	1	Coiling right	No
25	51	Fem	N	84	Bilat	AcomA	1	Clipping left	Left
26	55	Fem	N	4	Bilat	PcomA	3	Coiling right	No
27	43	Mal	N	24	Bilat	AcomA	1	Coiling left	No
28	55	Fem	Y	60	Bilat	AcomA	2	Coiling bilat.	No
29	31	Fem	N	48	Right	AcomA	2	Coiling bilat.	No
30	52	Fem	N	48	Bilat	PcomA	4	Clipping left	Left
31	46	Fem	N	12	Bilat	MCA	4	Clipping/Coiling right	Right
32	43	Fem	N	24	Bilat	MCA	3	Clipping/Coiling right	Right
33	46	Fem	N	12	Bilat	PcomA	3	Coiling bilat.	No
34	74	Fem	N	12	Bilat	AcomA	2	Clipping/Coiling left	Left
35	36	Fem	N	12	Left	AcomA	2	Clipping/Coiling right	Left
36	56	Fem	N	48	Right	MCA	3	Clipping right	Right
37	46	Fem	N	24	Bilat	AcomA	2	Clipping/Coiling right	Right
38	46	Fem	Y	48	Right	PcomA	4	Clipping right	Right
39	58	Fem	Y	5	Left	AcomA	2	Coiling bilat.	No
40	64	Fem	Y	4	Right	AcomA	4	Clipping right	Right
41	44	Fem	Y	12	Bilat	MCA	2	Clipping right	Right
42	62	Mal	Y	48	Vary	PcomA	4	Clipping left	Left
43	41	Fem	Y	12	Vary	MCA	2	Clipping left	Left
44	48	Fem	Y	48	Bilat	MCA	2	Clipping right	Right
45	39	Mal	Y	72	Bilat	ACA	4	Clipping left	Left
46	49	Mal	Y	60	Vary	AcomA	2	Clipping right	Right

*Age, age at the time of subarachnoid hemorrhage (SAH); y, history of migraine aura; n, no history of migraine aura; Migr.Atc/Y, Episodes the year before bleeding fulfilling the criteria of a migraine headache attack (isolated aura not included); Dom. Side, Dominant side/location of the migraine headache; Aneurysm Localization, intracranial artery of the bleeding aneurysm (ICA, internal carotid artery; MCA, middle cerebral artery; ACA, anterior cerebral artery Side of Trepanation: if performed; PCA, posterior cerebral artery; AcomA, anterior communicating artery; PcomA, posterior communicating artery); Hunt and Hess, grade on scale, Side of Trepanation: if performed*.

**Table 3:** Migraine situation prior to subarachnoid hemorrhage (SAH) and long-term course following SAH.

**#**	**pre** **SAH**	**post** **SAH**	**Comparison** **pre vs. post**	**Trepanation/ Side**	**Outcome** **(BI/mRS)**	**Correlation**
1	24	0	+ (!)	Right	100/1	x
2	12	0	+ (!)	Center	100/1	x
3	18	0	+ (!)	Right	95/1	x
4	6	0	+ (!)	Right	100/1	x
5	36	0	+ (!)	Right	100/1	+
6	30	0	+ (!)	Right	100/1	+
7	12	0	+ (!)	Right	95/2	+
8	12	4	+	Right	100/1	+
9	4	0	+ (!)	Right	100/1	+
10	24	0	+ (!)	Left	90/3	0
11	48	0	+ (!)	Left	100/1	x
12	12	0	+ (!)	Left	100/1	0
13	12	12	-	No	100/0	n
14	42	0	+ (!)	No	100/1	n
15	72	48	-	No	100/0	n
16	12	0	+ (!)	No	100/2	n
17	10	5	-	No	100/0	n
18	12	0	+ (!)	No	100/0	n
19	12	0	+ (!)	Right	75/2	+
20	4	1	+	Left	100/1	x
21	48	0	+ (!)	Left	100/1	x
22	18	4	+	No	100/0	n
23	24	0	+ (!)	No	100/0	n
24	6	3	+	No	100/1	n
25	84	0	+ (!)	Left	100/1	x
26	4	0	+ (!)	No	100/2	n
27	24	0	+ (!)	No	100/1	n
28	60	60	-	No	100/0	n
29	48	1	+	No	100/0	n
30	48	0	+ (!)	Left	100/2	x
31	12	0	+ (!)	Right	100/2	x
32	24	0	+ (!)	Right	90/2	x
33	12	0	+ (!)	No	100/1	x
34	12	0	+ (!)	Left	100/0	x
35	12	4	+	Left	100/0	+
36	48	0	+ (!)	Right	100/1	+
37	24	0	+ (!)	Right	100/1	x
38	48	4	+	Right	90/3	+
39	5	5	-	No	100/1	n
40	4	0	+ (!)	Right	100/0	+
41	12	0	+ (!)	Right	100/1	x
42	48	0	+ (!)	Left	35/4	x
43	12	12	-	Left	100/2	x
44	48	12	+	Right	100/2	x
45	72	0	+ (!)	Left	100/2	x
46	60	3	+	Right	100/3	x

*pre SAH/post SAH: Episodes per year fulfilling the criteria of a migraine headache attack (isolated aura not included) before bleeding, Comparison pre vs. post: +: Significant reduction (≥50%) / + (!): Complete disappearance / -: No significant reduction, Trepanation /Side: Side of trepanation if performed, Outcome: BI: Barthel index / mRS: modified Rankin scale, Correlation: +: Side of Pain and Trepanation equal, significant reduction of migraine / -: Side of Pain and Trepanation equal, NO significant reduction of migraine / 0: Side of Pain and Trepanation NOT equal / x: No dominant pain side / n: No trepanation*.

During an observation period of 46 months (mean, range 12–114 months, SD ± 27.8), migraine attacks stopped in 31 out of 46 patients (67.4%). After craniotomy, 24 (77.4%) patients had no further attacks of migraine headache and six (19.4%) showed a reduction of >50%, while only one (2.2%) did not report any relevant change. Without craniotomy, seven patients (46.7%) had no further migraine attacks and three (20%) had a reduction >50%, but no change was noted in five (33.3%) patients. Complete remission of migraine attacks was significant in both groups, the craniotomy (*p* < 0.001) and the no-craniotomy group (*p* = 0.002, Wilcoxon test). Besides, the number of patients reporting a reduction in migraine attacks of at least >50% compared to the time prior to SAH occurred in both groups (craniotomy: *p* < 0.001, no-craniotomy: *p* = 0.001, Wilcoxon test). Following craniotomy, patients showed a significantly higher rate of reduced migraine attacks (either complete or incomplete remission, *p* = 0.01, two-tailed Fisher exact test) and complete remission (*p* = 0.049, two-tailed Fisher exact test), respectively. For the other migraine criteria, we observed a significant decrease in the duration (*p* < 0.001) and intensity (*p* < 0.001) following SAH in both groups whereas the character of pain or concomitants (e.g., photophobia or nausea) did not change significantly.

A total of 11 out of 31 patients who underwent craniotomy reported a unilateral headache and in nine of them, surgery was performed on the corresponding side. All 11 reported a complete disappearance of migraine following SAH. Twenty of the surgical patients suffered from bilateral migraine pain or reported varying lateralization of headache. Of those, 16 were free of further migraine attacks. Regarding the pre-bleeding presence of migraine with or without aura, there was no statistical difference (*p* = 0.17, Fisher exact test).

A total of 16 out of 46 patients (34.8%) reported other types of headaches before SAH that clearly did not meet the migraine criteria. Thirteen cases (28.3%) fulfilled the IHS criteria for tension-type headaches (TTH). In three cases (6.5%), we diagnosed unspecific headaches. After treatment, TTH and unspecific headaches increased to 32.6 and 10.9%, respectively.

To test for potential confounders, such as arterial hypertension, diabetes, consumption of alcohol and nicotine, and medication containing hormones, we compared the status before and after SAH for the 31 patients with completely suspended migraine and the 15 patients with ongoing migraine. There was no significant difference in a before-after comparison. In addition, we matched both groups referring to age, sex, migraine aura, the Hunt and Hess (grade I-IV) score, Fisher grade (grade I-IV), the occurrence of hydrocephalus, and changes in medication used for first- or second-line prophylaxis in migraine (e.g., metoprolol, propranolol, flunarizine, topiramate, valproate, tricyclic antidepressants, gabapentin, or mirtazapine). No significant difference was detected regarding age (*p* = 0.13, Mann–Whitney-U test) and sex (*p* = 1.0, Fisher exact test). The mean age was 48.9 ± 9.9 years (5 men, 26 women) in the group without migraine compared to 44.9 ± 7.4 years (3 men, 12 women) in the group with ongoing migraine. A significant difference was found only in relation to the Hunt and Hess scores, as migraine disappeared significantly more frequently in patients with higher Hunt and Hess levels (2.81 vs. 2.1, *p* = 0.016, Mann–Whitney test). Fisher grade (*p* = 0.98) and aura (*p* = 0.165) did not show a significant difference.

## Discussion

The burden of migraine is impressively reduced after acute SAH despite the treatment procedure. During a follow-up period of 12–114 months (mean 46 ± 26.8 months), 40 out of 46 patients had a significant reduction in headache frequency, 31 even reported a complete cessation of migraine symptoms. The reduction of migraine headaches following SAH, however, occurred significantly more often in patients following craniotomy. Regarding the possible role of the MMA, this is consistent with the small studies of Dickerson (10) and Fan et al. (11). They reported a dramatic improvement in headache frequency in a long-term follow-up for up to 14 years after ligation of the MMA. Yet, the comparability with our observation is limited. In the cohort of Dickerson and Fan et al., all the patients suffered from intractable or refractory migraine, and particularly in invasive interventions, a high ratio of placebo effects may be present (20, 21). On the contrary, we designed our study as an observational survey without the objective to influence the course of migraine. Therefore, a direct impact of meningeal vessels exceeding a sham effect may be considered (22–24). Reduction of dural circulation by occlusion of the MMA might be followed by a decrease of the pulse wave amplitude that usually stimulates the arterial wall nociceptors resulting in typical pulsating pain characteristics. It might also lead to the reduction of endothelial leaking and extravasation of plasma, respectively, inflammatory mediators by lower blood flow, blood pressure, and/or reduced vasodilation. An additional or independent explanation may be the transection of dural trigeminal nerve branches that follow meningeal arteries, which might affect migraine on a neural level. Vasoactive neuropeptides and pain modulators such as CGRP and PACAP originate from the trigeminocervical complex and reach the dural vessels *via* such nerve fibers (25).

The fact that not only patients with migrane undergoing a craniotomy but also those undergoing endovascular embolization showed a remission from migraine after SAH suggests that other factors also may be involved. It is known that patients with an unruptured intracranial aneurysm (UIA) revealed a significantly higher migraine prevalence of 24.4% compared to 14.6% in a control group (26). Thus, migraine frequency could decrease when this risk factor is eliminated, though the pathophysiologic basis remains unclear. A potential relation between migraine and UIA has also been highlighted by a case-control study, including 51 patients with UIA and a preceding migraine in the year before surgery (27). Interestingly, migraine was significantly reduced by 74.5% 1-year after clipping, which is comparable to our craniotomy subgroup treated by clipping (77.4%).

Our results support the assumption that the elimination of an intracranial aneurysm ameliorates migraine markedly. The long average migraine history of 23.9 years in our study argues, however, that migraine is likely not related to the presence of a UIA alone.

Getting older has a great influence on the long-term course of migraine frequency and severity up to complete remission at a higher age. The overall mean age was 47.6 ± 9.3 years in our study. It seems unlikely that the dramatic improvement of migraine complaints can be solely explained by aging processes during a mean follow-up period of almost 4 years.

Newly acquired headache after SAH especially of TTH appears to be a common phenomenon. After clipping of UIA, a nonsignificant increase of TTH was reported from 37.9 to 50.6% (27). Accordingly, in our study, we also noted a nonsignificant increase in TTH occurrence from 28.3 to 32.6%.

Several limitations must be mentioned. The demographic data and characteristics of migraine in our study are only partially consistent with the general population. In addition, our study population is not randomized, and findings lack a control group of either otherwise healthy patients with migraine or patients with SAH only. The loss to follow-up of 14 of the 60 subjects was rather high and could affect the results. Furthermore, the small sample size weakens the significance of comparing surgical vs. endovascular patients. There is also a potential for recall bias, as the pre-ictus migraine severity is determined retrospectively. In addition, the higher proportion of patients with a higher Hunt and Hess grade who have become symptom-free may be due to a limitation of their cognitive abilities. Nonetheless, the results seem worth further discussion and investigation. It would be especially interesting and could substantiate our findings to observe and compare the course of preexisting migraines in non-SAH patients who had a craniotomy performed for other reasons. Unfortunately, we did not find such data in the literature.

## Conclusion

Our data suggest that transection of the dura mater with the MMA and accompanying trigeminal branches may improve substantial migraine headache and not the elimination of the aneurysm alone. Further options of treatment should depend on future results of observational studies in patients with preexisting migraines who underwent a craniotomy.

## Data Availability Statement

The datasets presented in this article are not readily available because the participants of this research did not agree for their complete datasets to be shared publicly. Requests to access the datasets should be directed to JV, jose.valdueza@segebergerkliniken.de.

## Ethics Statement

The studies involving human participants were reviewed and approved by the Medical Association Schleswig-Holstein (Germany) AZ 020/12 (I) and Charité University Medicine Berlin (Germany) EA4/022/09. The patients/participants provided their written informed consent to participate in this study.

## Author Contributions

JV conceived of the presented idea. JV, VW, and JD further developed the theory and performed analysis. All authors collected data at the different locations, discussed the results and contributed to the final manuscript. All authors contributed to the article and approved the submitted version.

## Funding

The work was financially supported by grants of the ERA-NET NEURON Network of European Funding for Neuroscience Research EBio2 and Deutsche Forschungsgemeinschaft (DFG) DFG DR 323/10-1 to JD; FP7 no 602150 CENTER-TBI to JD and OS; DFG DR 323/5-1 to JD, JW, and OS; DFG WO 1704/1-1 to JW. There was no involvement of the funding sources regarding study design, data collection, analysis, interpretation, writing, or decision of publication.

## Conflict of Interest

The authors declare that the research was conducted in the absence of any commercial or financial relationships that could be construed as a potential conflict of interest.

## Publisher's Note

All claims expressed in this article are solely those of the authors and do not necessarily represent those of their affiliated organizations, or those of the publisher, the editors and the reviewers. Any product that may be evaluated in this article, or claim that may be made by its manufacturer, is not guaranteed or endorsed by the publisher.
